# Rare tumor in unusual location – glomus tumor of the finger pulp (clinical and dermoscopic features): a case report

**DOI:** 10.1186/s13256-018-1721-0

**Published:** 2018-07-08

**Authors:** Ghita Senhaji, Salim Gallouj, Ouiame El Jouari, Amina Lamouaffaq, Mouna Rimani, Fatima Zahra Mernissi

**Affiliations:** 1 Department of Dermatology, University Hospital Hassan II, Fez, Morocco; 2 Hassan Center of Anatomopathology, Rabat, Morocco

**Keywords:** Glomus tumor, Case report, Dermoscopy, Histology, Rare form, Pulp

## Abstract

**Background:**

Glomus tumors are rare, benign, vascular neoplasms arising from the glomus body. Although they can develop in any part of the body, they commonly do so in the upper extremities, most frequently subungual areas. They can be either solitary or multiple. Most typically they present as a small, round, bluish nodule visible through the nail plate with a classic triad of symptoms: hypersensitivity to cold, heightened pinprick sensitivity, and paroxysmal pain. Several tests can help in diagnosing these tumors with multiple imaging tools such as X-ray, magnetic resonance imaging, and ultrasonography. However, only histology can confirm the diagnosis. Complete surgical excision of the tumor is the only effective treatment to achieve pain relief and low recurrence rate.

**Case presentation:**

We report here a rare case of a 54-year-old Moroccan Berber woman presenting with a 10-year history of a glomus tumor at an unusual site. Dermoscopy and histology were helpful to confirm the diagnosis.

**Conclusions:**

We aim to discuss clinical, dermoscopical aspects of this tumor and surgical modalities. We also emphasize the importance of keeping this tumor in mind among the possibilities of differential diagnosis of painful digital nodules.

## Background

Glomus tumors, as first described by Masson in 1924, are rare, benign, vascular neoplasms arising from the glomus body, which is a contractile neuromyoarterial structure found in the reticular dermis [1], responsible for adjusting blood pressure and temperature by regulating blood flow within the cutis [2]. Although they can develop in any part of the body, they commonly do so in the upper extremities, most frequently in subungual areas [3]. Approximately 10% of these tumors occur on the pulp of the distal phalanx [4]. The classic triad of symptoms, paroxysmal pain, localized hyperalgesia, and sensitivity to cold temperature are important diagnostic features [5]. Clinical features are little known, which explains a frequent diagnostic delay. The contribution of radiological assessment is discussed, the diagnostic being confirmed by histology [6]. We report here a rare case of a glomus tumor located in an uncommon location, with the patient presenting a typical triad of symptoms. To the best of our knowledge, this is the first case that presents dermoscopical aspects of this tumor in such a rare location.

## Case presentation

A 54-year-old, right-handed unemployed Moroccan Berber woman from an urban area reported a personal medical history of intermittent epigastric pain without a history of diabetes or chronic disease, nor any special psychosocial background, and with a familial history of allergic rhinitis. She presented with a 10-year history of progressively intense pain, cold sensitivity, and severe tenderness to palpation of the pulp of her left little finger, with no gross abnormalities of her fingers, and no previous trauma history. The pain increased when her digit was exposed to cold. Furthermore, the tip was excessively sensitive to touch, and her pain increased at night. She had seen a primary care doctor, with no definitive diagnosis. Moreover, she reported occasional intake of omeprazole for intermittent abdominal pain. She had no history of active or passive tobacco smoking or alcohol intake. She also had no past intervention. She was referred to our department for surgical excision with histopathological examination.

**Fig. 1:**
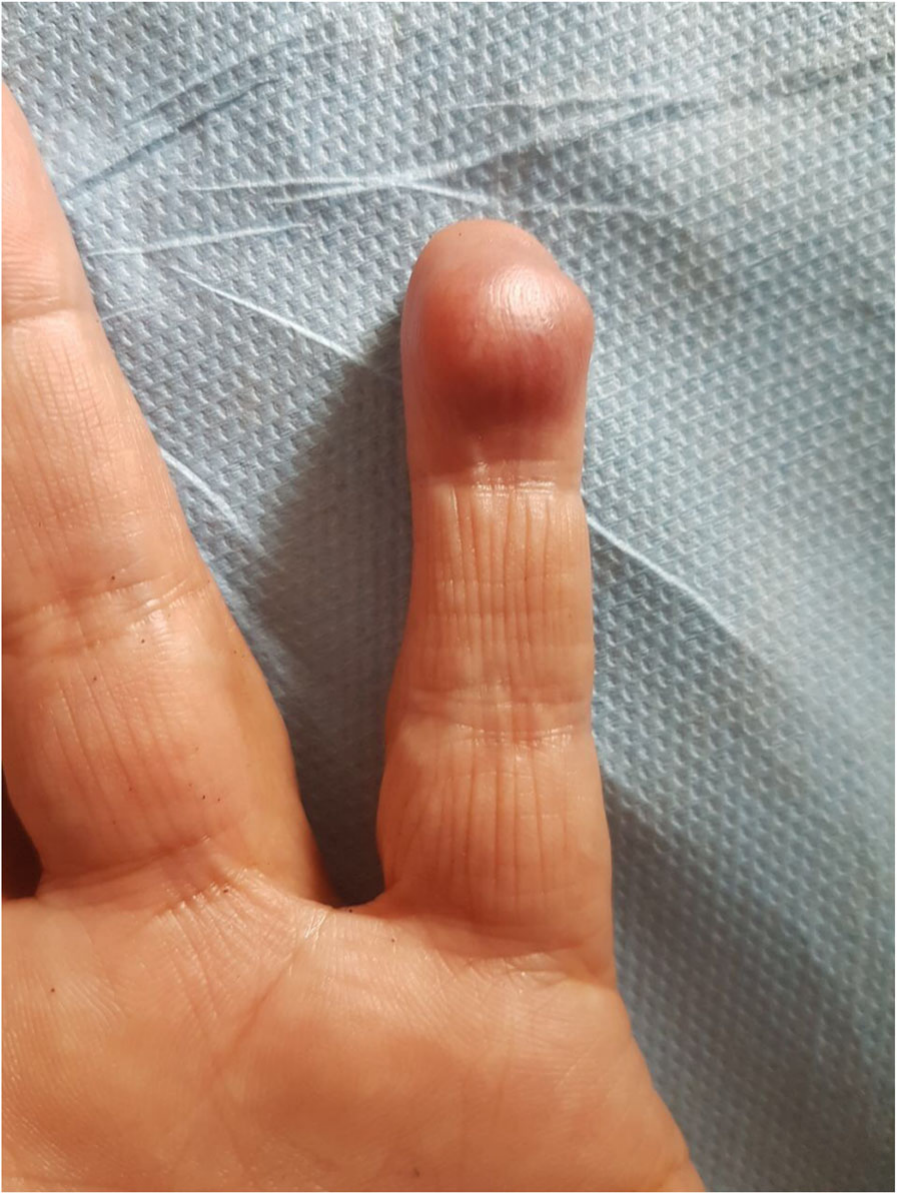
Subcutaneous nodule of approximately 1.5 cm, of firm consistency and pinkish red coloration, streaked by multiple telangiectasias on the pulp of the left finger

**Fig. 2:**
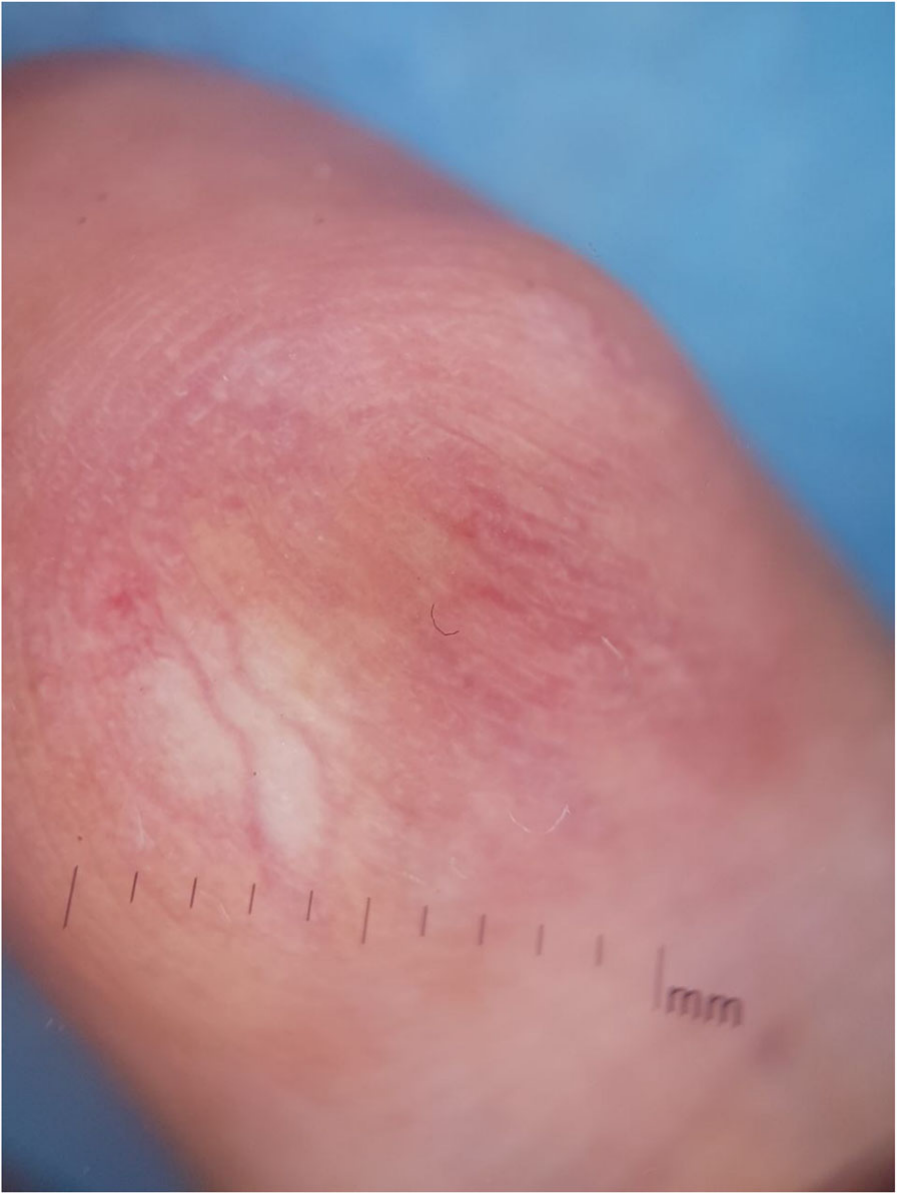
Polarized, contact dermoscopy showing peripheral clearing of the reddish color, disclosing a yellow to white background, with multiple telangiectasias

A clinical examination showed a well-oriented, apyretic, and eupneic patient, with normal cardiac frequency and regular blood pressure, presenting with a painful subcutaneous nodule of approximately 1.5 cm, of firm consistency and pinkish red coloration, streaked with multiple telangiectasias on the pulp of the distal phalanx of her left little finger (Fig. 1). Polarized contact dermoscopy induced peripheral clearing of the reddish color, disclosing a yellow to white background, with multiple telangiectasias on the surface (Fig. 2). A neurological examination showed no signs of paresthesia or hypoesthesia in the area of the tumor, nor at a distance, with a preserved muscular and neurological function; a general examination showed no other abnormality.

**Fig. 3:**
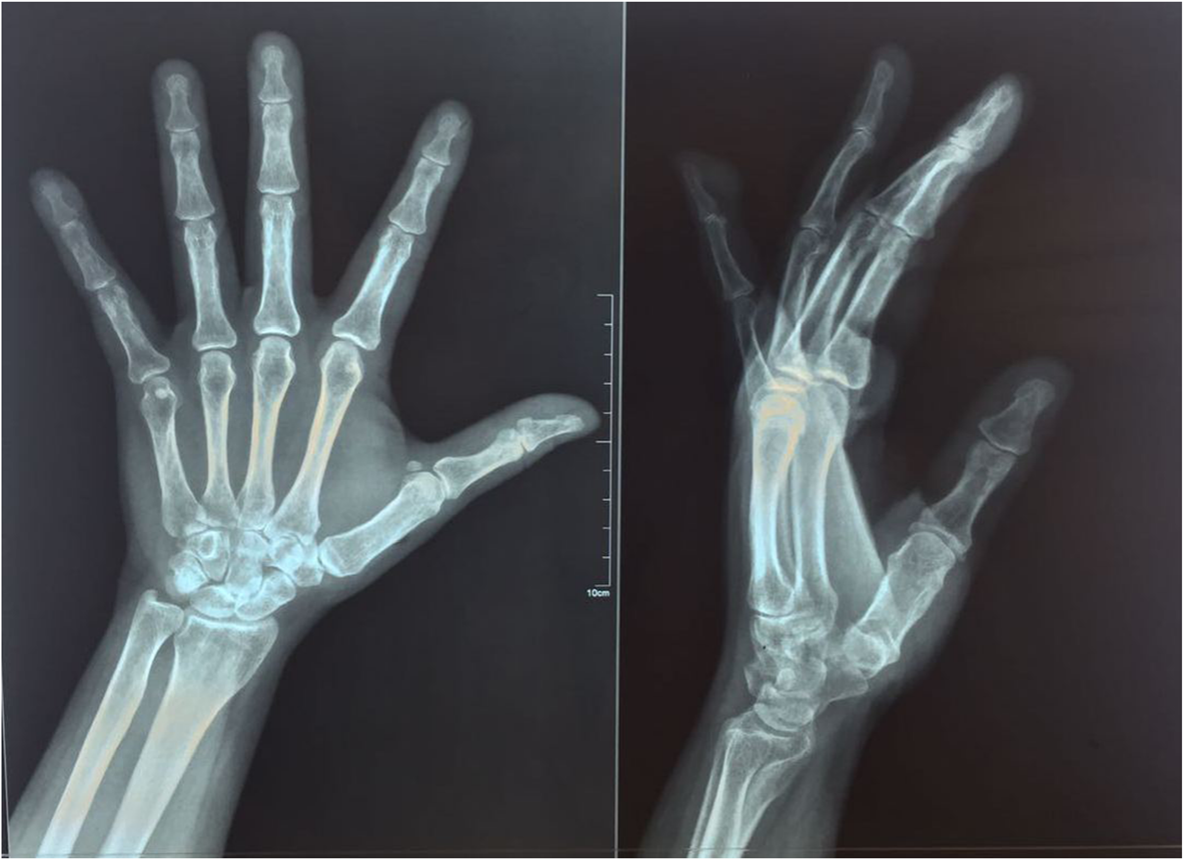
Radiograph showing a mass on the soft tissue of the distal pulp of the patient’s left little finger with no bony erosion

The differential diagnosis at the time of examination included glomus tumor, schwannoma, mucoid cyst, and neurofibroma. An X-ray study was done for her left hand. No bony lesions were identified by radiographic studies (Fig. 3). She did not benefit from ultrasonographic imaging or magnetic resonance imaging (MRI) because the diagnosis of glomus tumor was highly probable.

**Fig. 4:**
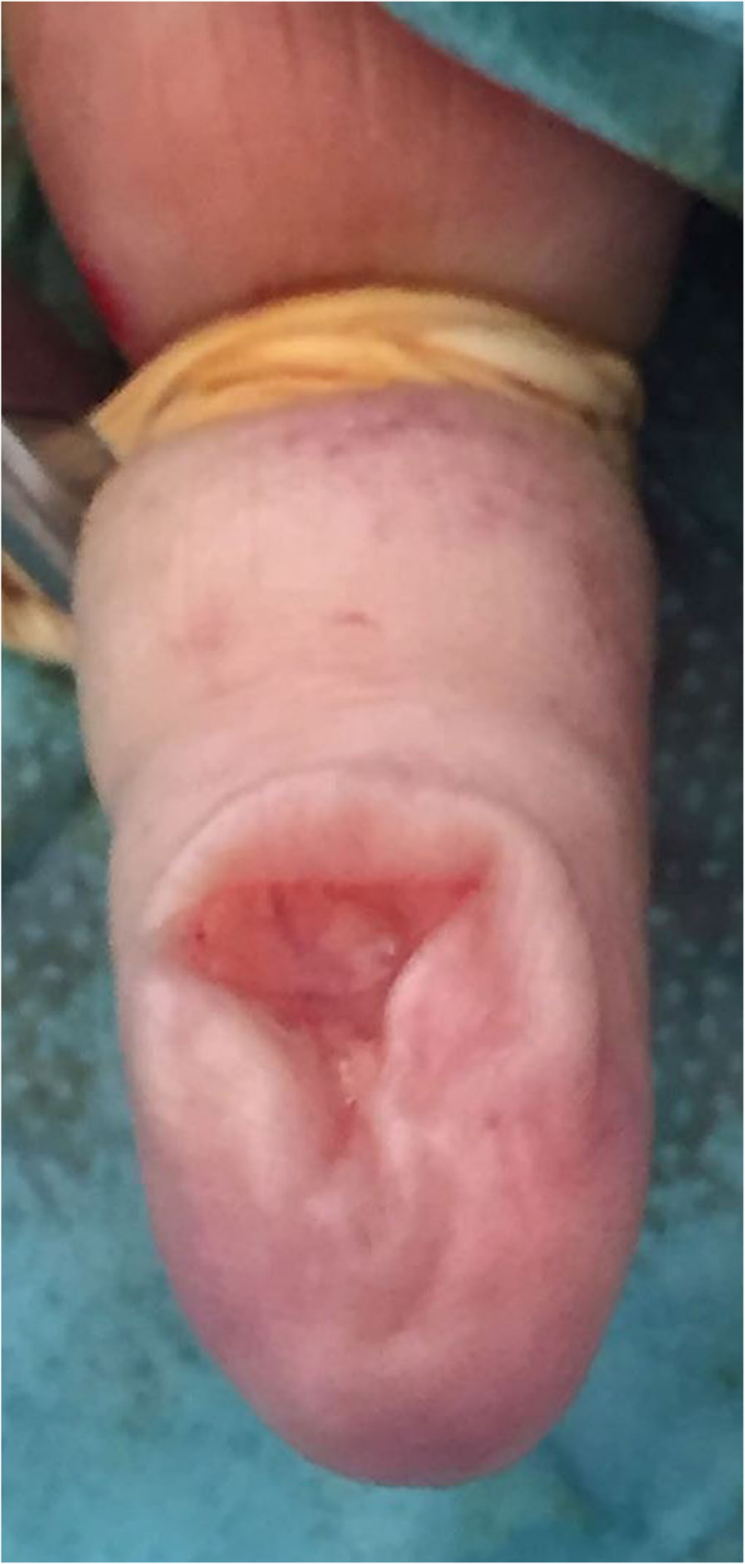
Intraoperative photograph of the lesion showing tumor resection with a volar approach

**Fig. 5:**
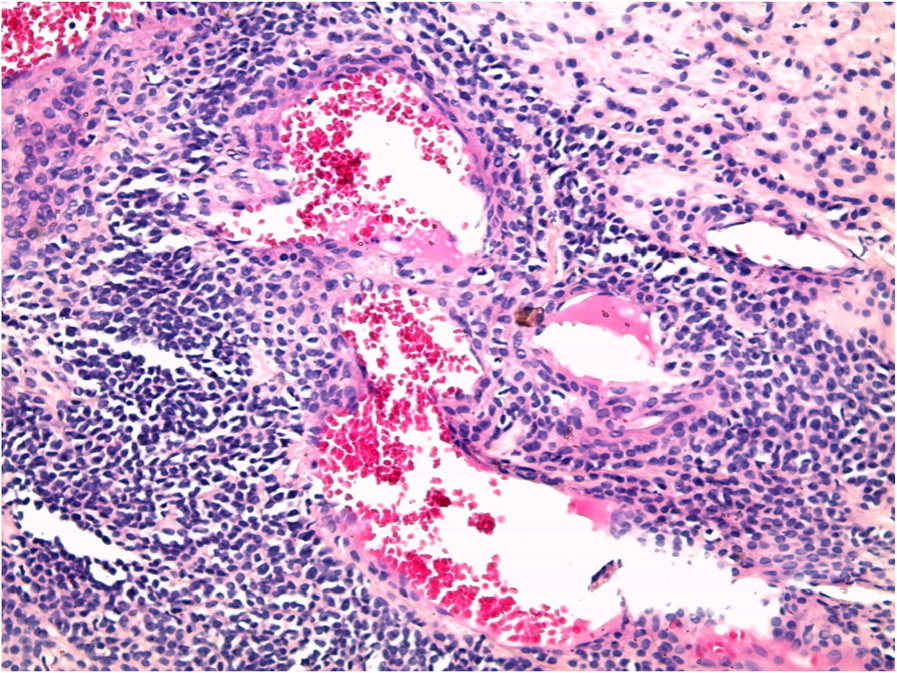
Histological section of the tumor. Proliferation of typical glomus cells arranged in cords and blocks in greater increase (hematoxylin and eosin staining, × 200)

Surgical intervention was performed. A paramedian volar incision was made of the pulp of the distal phalanx of her left little finger. The mass was well circumscribed and removed with blunt dissection and sent to pathology (Fig. 4). It was a red soft tissue nodule of 1.5 cm in diameter and had no stalk or adherences to a joint. It was removed completely and dermoscopy of the excised tumor was performed showing yellow structureless areas surrounded by linear vessels. A histological examination confirmed a glomus tumor showing a tumor proliferation arranged around many narrow vascular clefts that circumscribed flattened endothelial cells. These vessels were surrounded by several superimposed layers of ovoid cells with round, regular nuclei and moderately acidophilic cytoplasm with imprecise boundaries (Fig. 5). In places, these elements deviated from the vascular walls and spread irregularly, sometimes isolated or in small clusters, within a fibromyxoid stroma strewed with lymphocytes and some plasma cells. Her symptoms improved on removal of tumor and she healed without complication. At follow-up visits, she presented no signs of recurrence with complete healing of the pain within 1 year.

## Discussion

Here we present a rare case of a histologically confirmed glomus tumor of the pulp, which is an unusual site of such tumors, with a 10-year history of intense pain, cold sensitivity, and severe tenderness to palpation of the pulp. Through this case, we emphasize the dermoscopical features of this tumor in such a location, which are lacking in the literature, and we detail histological aspects and operative procedure.

These tumors, also known as Barré–Masson syndrome, are rare benign hamartomas that arise from the normal glomus apparatus, located in subcutaneous tissue [4]. They were first reported by Wood in 1812 [2], but were not attached to a glomic origin until 1924 by Masson [6]. Glomus bodies are present throughout the body but highly concentrated in the tips of digits, especially under the nail [6]. So, the tumors are usually in the subungual area [4]. Thus, finding a glomus tumor in the pulp of the distal end of our patient’s finger, as presented here, instead of underneath the nail bed, is unusual [7].

The etiology of glomus tumors is unknown and it may be related to sex, age, trauma, or inheritance. Some authors have proposed that a weakness in the structure of a glomus body could lead to reactive hypertrophy after trauma [1]. Such risk factors were not reported by our patient.

It has a variable evolution time between days and decades; it is a difficult tumor to diagnose due to its rarity, which justifies long delays in diagnosis and the establishment of therapy [5].

Glomus tumors are categorized as either solitary or multiple, according to their clinical presentation [3]. The most frequent is the solitary type, which affects more females in middle age, as in the present case, with predominant location on the fingers, more commonly in the distal phalanges [5]. Of all glomus tumors, 75% are subungual in location [1]. The pulp of the distal phalanx is a very rare location for a glomus tumor [4]. It appears as a small, slightly raised, bluish or pinkish red, painful nodule [1], as seen in our patient.

Although the cause of pain in glomus tumor is not clearly understood, several hypotheses have been proposed [1]: the capsules of the tumors render them sensitive to pressure; abundant mast cells in the glomus tumors release substances such as heparin, 5-hydroxytryptamin, and histamine, causing receptors to pressure or cold stimulation to be sensitive [3]; and excessive dominance over the nerve of numerous non-myelinated nerve fibers that penetrate into glomus tumors [1].

The dermoscopic appearance of glomus tumors can be very subtle. In subungual tumors, nail plate dermoscopy can find the presence of linear vascular structures [8], whereas extradigital tumors can reveal a homogeneous white structure and peripheral telangiectasias [9]. However, quite often these structures can be discrete or absent. Diagnosis can, therefore, be easily missed [8]. To the best of our knowledge, this is the first case reporting the dermoscopic features of a finger pulp glomus tumor.

Importantly, the diagnosis of glomus tumor must be made through the history and clinical examination of a patient [4]. Typically, it manifests with a classic triad of symptoms: hypersensitivity to cold, heightened pinprick sensitivity, and paroxysmal pain [7]. Other symptoms include distinctive subungual discoloration, hypoesthesia, atrophy, osteoporosis in the lesion, and autonomic disturbance such as Horner syndrome [3].

In addition to the classical presentation, there are three useful tests that help in diagnosing these tumors [1]: Love’s pin test, Hildreth’s test, and cold sensitivity test [7]. The cold sensitivity test was positive in our case, which confirmed the diagnosis of a glomus tumor. Additional tests such as a simple X-ray, computed tomography (CT), angiography, and ultrasonography can be conducted for more accurate diagnosis [3]. Radiographs can show cortical thinning or erosive changes in the adjacent bone in some cases [1]. Our patient had no such abnormalities. MRI can also be used; it is noninvasive and it provides excellent contrast between a neoplasm and normal tissue [3], showing a high-signal nidus surrounded by a rim of lower signal intensity [2]. It can also be helpful in making differential diagnoses, such as neuroma, melanoma, pigmented nevus, and hemangioma, as well as foreign bodies [1]. Ultrasonography can be a better option than MRI, considering the time required for the test, its cost, and its ability to enable the evaluation of lesions dynamically in real time [3]. It shows commonly hypoechogenic lesions up to 3 mm in diameter [5].

In this case, X-rays revealed the mass to be in the distal pulp of our patient’s little finger. Further radiological investigations were not necessary in our patient because of the clinical features of the tumor and the presence of paroxysmal pain exacerbated by cold.

Histopathological analysis reveals variable composition of glomus cells, blood vessels, and smooth muscles [1]. It may show a neoplasm composed of polygonal cells, with small and regular nucleus, sometimes in solid clusters, or in regularly oriented cellular cords [5]. Glomus cells are organized in nests around vessels [6], which were seen in our case.

Although glomus tumors are essentially benign, sarcomas accompany benign glomus tumors in rare cases to form glomangiosarcoma [3]. Histology can also rule out the diagnosis of malignant melanoma [6].

Solitary glomus tumors need to be ruled out from painful tumors, such as leiomyoma or eccrine spiradenoma [3]. Moreover, painful tumors such as hemangioma, neuroma, or gouty arthritis can simulate a glomus tumor in the hand leading to a diagnostic enigma and can pose a therapeutic challenge [1].

Complete surgical excision of the tumor is the only effective treatment [1]. For subcutaneous or pulpal tumors, the approach is direct, respecting the principles of cutaneous incisions and avoiding nerve fiber pathways [6]. In our case, the direct approach was sufficient for complete excision because the lesion was located in the pulp. In all cases, total extirpation is performed after carefully avulsing the surrounding tissues of the tumors [3]. After complete tumor removal, pain relief is rapid and the finger regains its normal appearance in 3 months [6]. If not, re-exploration of the affected area and repeat imaging should be done [1].

Meticulous care needs to be taken at the first operation to completely remove all lesions [3] because the recurrence rate can be from 5 to 50%, mainly due to incomplete excision [7].

## Conclusions

We report the case of a glomus tumor arising in the unusual location of the pulp of a finger, with typical symptoms of long-term pain and sensitivity to touch. Here we are the first to describe the dermoscopic features of a glomus tumor in the rare location of the pulp of a finger. We aim to emphasize the importance of the inclusion of the glomus tumor among the possibilities of differential diagnosis of painful digital nodules, despite its low occurrence. Clinicians should also keep the possibility of these tumors in mind and perform careful examinations and preoperative tests. Complete surgical excision is mandatory to get complete relief from the symptoms and to avoid recurrence.
